# Dispersal route of the Asian house rat (*Rattus tanezumi*) on mainland China: insights from microsatellite and mitochondrial DNA

**DOI:** 10.1186/s12863-019-0714-3

**Published:** 2019-01-22

**Authors:** Song Guo, Guichang Li, Jinli Liu, Jun Wang, Liang Lu, Qiyong Liu

**Affiliations:** 1 State Key Laboratory of Infectious Disease Prevention and Control, National Institute for Communicable Disease Control and Prevention, Chinese Center for Disease Control and Prevention, Beijing, 102206 China; 2 Zhejiang Center for Disease Control and Prevention, Hangzhou, 310051 China

**Keywords:** *Rattus tanezumi*, Population genetics, Dispersal, Mainland China

## Abstract

**Background:**

*Rattus tanezumi* is a common commensal rat and an important host animal of bubonic plague in South China and Southeast Asia. The northward dispersal of this species in mainland China has been reported in recent decades, along with more recent intercontinental expansion. Population genetics of *R. tanezumi* in mainland China were studied to explain the relationship between dispersal history and the ancient and modern transportation networks of China.

**Results:**

In total, 502 individuals belonging to 18 populations were collected from 13 provincial areas. Nine microsatellite loci and two mtDNA sequences were analyzed. The results indicate that *R. tanezumi* populations from Yunnan have highest genetic diversity and populations from Tibet with lowest genetic diversity. 18 populations can be divided into four clusters, the first cluster including populations from southwest Yunnan, the second including two populations of Tibet, the third for populations from middle and east of mainland China, and the forth for two populations from north Yunnan. Both microsatellite and mtDNA data reveal that the populations from coastal areas are closely related to populations from Yunnan, whereas populations from Tibet are closely related with populations from Sichuan.

**Conclusions:**

The results suggest that early dispersal of *R. tanezumi* in mainland China depended on shipping transportation, with subsequent expansion from coastal areas into Central China occurring along the Yangzi River. Further, the linkages between populations in Tibet and Sichuan point to a modern era introduction via the Chuan-Zang highway, rather than along the Tea Horse Ancient Road.

**Electronic supplementary material:**

The online version of this article (10.1186/s12863-019-0714-3) contains supplementary material, which is available to authorized users.

## Background

The Asian house rat, *Rattus tanezumi* (Temminck, 1844) (Rodentia: Muridae), is a common commensal rat in East and Southeast Asia. From South China to Southeast Asia, *R. tanezumi* can be found in indoor and outdoor habitats. In outdoor habitats, it is the dominant pest rodent in rice fields, causing crop damage before harvest [1, 2]. After the harvest season, *R. tanezumi* will migrate to residential areas and even indoor areas for food [3], causing household damage [4]. Because of its wide range of habitats and these seasonal migrations, *R. tanezumi* can carry a number of different pathogens including zoonotic agents of public health concern. Thus, *R. tanezumi* is regarded not only as a pest related to agriculture but also as a host of several zoonoses, including plague (*Yersinia pestis*), haemorrhagic fever with renal syndrome (*Hantaan* virus), and leptospirosis (*Leptospira*) [5–7].

The range expansion of *R. tanezumi* was not noted until recently after Huang et al. reported the northern border of the distribution area of *R. tanezumi* in China as the Yellow River [8]. From the 1990s on, *R. tanezumi* was found in provinces north of the Yellow River, including Hebei [9] and Shanxi [10], and in 2012 Ma et al. reported the first record of *R. tanezumi* northeast of Qinghai, a northwestern province of China [11]. The colonization of *R. tanezumi* in north China was easily recognized because this species can be distinguished from another commensal rat, *R. norvegicus*, with morphological characters. However, the expansion of *R. tanezumi* out of Asia was not recognized until genetic methods were used in congeneric species identification [12] because of the morphological similarities among members of the *R. rattus* species group. With molecular genetic methods, Bastos et al reported the presence of *R. tanezumi* in 13 locations in South Africa [13]. Mitochondrial haplotypes of *R. tanezumi* were also found in California in the United States and in Madagascar [14]. Based on these and additional data, Aplin et al inferred the prehistory expansion and the global distribution of *R. tanezumi* [15].

The extension of the geographic range of commensal rats is considered a result of human activity. The phylogeography of *R. rattus* in the western Indian Ocean is congruent with human colonization events in this area [16]. The distribution of *R. exulans* is also a good example and has been used to imply the history of human colonization on Pacific islands [17, 18]. *Rattus norvegicus* was not reported at Xinjiang before the 1970s because of the Gobi Desert between Xinjiang and Central China. In the 1970s, colonies of *R. norvegicus* were first found in railway stations of Xinjiang, after the railway was built [19]. From then on, *R. norvegicus* spread to other cities of Xinjiang with railway and highway traffic. Because the dispersion of *R. tanezumi* was reported only in recent decades, the dispersal routes of this species and how these relate to human activity is not known.

**Fig. 1:**
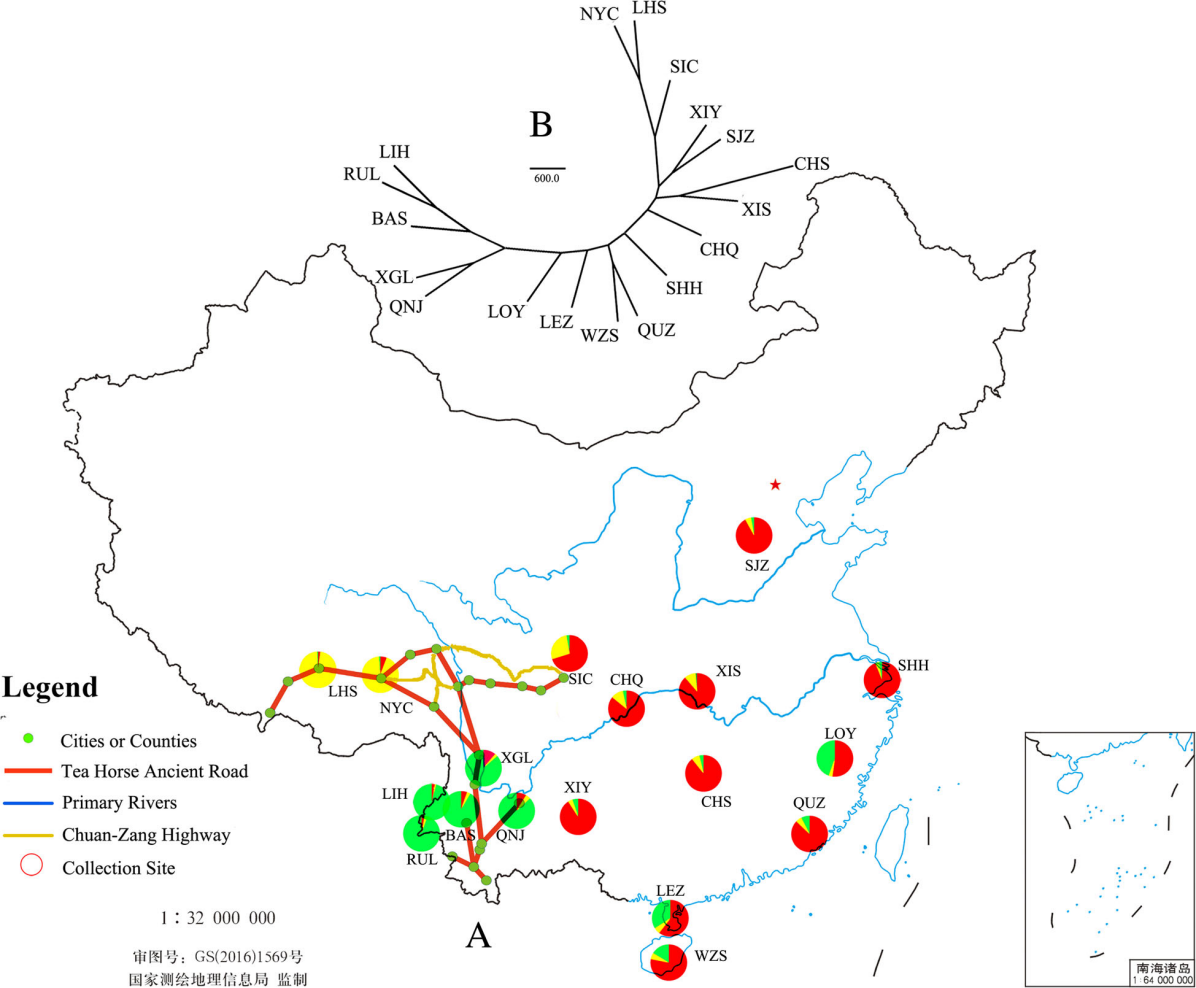
Sampling locations and genetic clusters of different populations used in this study (**a**) and the dendrogram based on microsatellites using Nei’s genetic distance (**b**). The map of China shows the locations of sampled rat populations, the Tea Horse Ancient Road, the Chuan-Zang Highway, and primary rivers in China. The coloured pie charts represent distinct genetic clusters found via Bayesian clustering analysis for each population, with red representing cluster I, yellow presenting cluster II and green for cluster III. The map is downloaded from the website of National Administration of Surveying, Mapping and Geoinformation of China (http://bzdt.nasg.gov.cn/index.jsp)

In ancient China, because of the complex landscape of Southwest China, commercial transportation in this area (including Sichuan, Yunnan, Shaanxi and Tibet) usually depended on caravans. Starting approximately 1200 years ago (Tang Dynasty), a famous caravan road system for tea, salt and horse trading developed in Southwest China, which is now called the Tea Horse Ancient Road. This road system linked Sichuan, Yunnan and Tibet (Fig. 1a) and became a unique route for trade between Tibet and Central China [20]. Another important transportation system in ancient China was shipping via sea, river and canal. Shipping transportation has been used on the Yangzi River and Pearl River for more than two thousand years. During the twentieth century, a modern highway system was gradually constructed in Southwest China, and modern transportation based on the automobile and highway system became a primary method of commodity trade. An important highway linking Tibet and Central China is the Chuan-Zang Highway, which starts from Chengdu, the capital city of Sichuan, and ends at Lhasa in Tibet (Fig. 1a). Whether and how these transportation systems affected the migration of *R. tanezumi* on mainland China, especially from Yunnan to Tibet, could be explained by population genetics analysis of rats.

In this study, we collected samples of *R. tanezumi* from mainland China and examined the genetic structure of different populations with microsatellite and mitochondrial markers. Based on the results of these analyses, we aimed to explain the expansion of *R. tanezumi* through mainland China, the possible expansion routes of *R. tanezumi* through mainland China, including the Tea Horse Ancient Road for caravans, shipping transportation along the rivers and coastline of China, and transportation along modern highway and railway systems.

## Methods

### Sampled populations and DNA extraction

Samples belonging to 18 populations were collected at 22 locations (*N* = 10–39 rats per location) in 13 provinces covering almost the entire range of *R. tanezumi* in China (Fig. 1a and Table 1). Samples of four populations (SHH, CHQ, SIC and QNJ) consisted of specimens pooled from two sites in close proximity. Rats were captured with living traps in rice fields and farms after getting verbal consents from owners. Individuals of *R. tanezumi* were selected and carried to the laboratory. Compressed carbon dioxide in gas cylinder was used to euthanize the rats. For the safety the flow rate of CO_2_ was controlled at 9 l per minute. Rats were euthanized in plastic bag with CO_2_ for ten minutes, and the death of them was confirmed with checking the heartbeat. Muscle or liver tissue was collected and stored in 95% ethanol at − 80 °C until DNA extraction according to the handbook of the DNeasy Blood & Tissue kit (Qiagen, GER).

**Table 1:** Information of populations included in the present research

Populations	Locality	Geographic coordinate	Altitude	Habitat	Sample size
LIH	Lianghe, Yunnan	24°53.61′N; 098°21.72′E	1000	Outdoor	30
RUL	Ruili, Yunnan	24°00.77′N; 097°51.11′E	760	Rice field	32
BAS	Baoshan, Yunnan	25°06.72′N; 099°09.69′E	1650	Rice field	29
XGL	Xianggelila, Yunnan	28°29.17′N; 098°54.69′E	1850	Indoor	16
QNJ	Qujing, Yunnan	25°29.40′N; 103°47.77′E	1866	Outdoor	17
	Jinning, Yunnan	24°40.18′N; 102°35.73′E	1900	Outdoor	15
LOY	Longyou, Zhejiang	29°18.41′N; 120°04.50′E	52	Indoor	26
LEZ	Leizhou, Guangdong	20°54.86′N; 110°05.81′E	20	Indoor	20
WZS	Wuzhishan, Hainan	18°46.51′N; 109°31.02′E	200	Indoor	33
QUZ	Quanzhou, Fujian	24°52.86′N; 118°40.32′E	20	Indoor	28
SHH	Putuo, Shanghai	31°14.97′N; 121°23.78′E	8	Indoor	20
	Pudong, Shanghai	31°11.04′N; 121°29.22′E	8	Indoor	30
CHQ	Wanzhou, Chongqing	30°48.47′N; 108°24.53′E	200	Indoor	29
	Yubei, Chongqing	29°43.09′N; 106°37.86′E	200	Indoor	11
XIS	Xingshan, Hubei	31°20.89′N; 110°44.81′E	200	Indoor	39
CHS	Changsha, Hunan	28°13.71′N; 112°56.42′E	50	Indoor	20
SJZ	Shijiazhuang, Hebei	38°02.54′N; 114°30.89′E	160	Indoor	18
SIC	Chengdu, Sichuan	30°39.52′N; 104°03.89′E	500	Indoor	12
	Qingchuan, Sichuan	32°35.06′N; 105°14.29′E	800	Indoor	10
XIY	Xingyi, Guizhou	25°05.52’N; 104°53.71′E	1160	Indoor	16
NYC	Nyingchi, Tibet	29°38.73′N; 091°08.56′E	2970	Indoor	21
LHS	Lhasa, Tibet	29°38.73′N; 091°08.56′E	3660	Indoor	30

### Microsatellite analysis

All samples were genotyped for 9 microsatellite loci developed for this research (Additional file 1: Table S1). DNA was amplified in a thermocycler (SensoQuest, GER) in 25-μL reaction mixes containing 1 μL of total DNA (about 30 μg), 2.5 μL of 10× LA PCR Buffer II (Mg^2+^ Plus) (Takara, JPN), 2.5 mM of each dNTP (Takara, JPN), 10 pmol of each primer, and 0.75 U of LA *Taq* polymerase (Takara, JPN). The 5′ end of the forward primer was labelled with a fluorescent dye (FAM, HEX, and ROX). Cycling conditions were as follows: 5 min at 95 °C, followed by 40 cycles of 30 s at 95 °C, 30 s at the annealing temperature (TA), and 30 s at 72 °C, and then 5 min at 72 °C. The length of the DNA fragments was analyzed manually in GeneMapper v. 4.0 (Applied Biosystems).

The genetic diversity of every locus was characterized by estimating number of different alleles (*N*_*a*_), number of effective alleles (*N*_*e*_), Shannon’s information index (*I*), number of private and locally common alleles, allele richness (*Rs*), observed heterozygosity (*H*_*o*_), unbiased expected heterozygosity (*H*_*e*_) and positive inbreeding coefficient (*F*_*IS*_) using Fstat V2.9.3.3 (https://www2.unil.ch/popgen/softwares/fstat.htm) and GenAlEx 6.41 [21]. Student’s *t*-Test was used to determine whether two sets of data were significantly different from each other in SPSS 16.0 [22]. Fstat was also used to test for conformance of the genotype to Hardy-Weinberg equilibrium (HWE) for each locus and each population and to explore linkage disequilibrium (LD) between pairs of loci. For all nine loci, Microchecker V2.2.3 [23] was used to detect the presence of null alleles and genotyping errors such as large allele dropout or stuttering using 1000 randomizations. The null-allele-adjusted dataset was compared to the original dataset to explore the effect of null alleles on estimate of genetics differentiation.

Isolation by distance (IBD) was estimated with Mantel’s test in Web Service of IBD [24], using the correlation between genetic and geographic distances by the regression of pairwise *F*_*ST*_/(1-*F*_*ST*_) on the natural logarithm (Ln) of straight-line geographic distance. Two approaches were used for testing genetic bottlenecks at the microsatellite loci for each population, we calculated M, a ratio based on the number of alleles to the allelic size range [25] in Arlequin3.5 [26], and heterozygosity excess tests implemented in Bottleneck V1.2.02 [27] to assess deviation from Mutation-Drift Equilibrium. The tests were performed using the Infinite Allele Model (IAM) [28], Stepwise Mutation Model (SMM) [29], and Two-Phase Model (TPM) [30], which were run twice for each population, assuming that the percentage of stepwise mutations was 80%. Statistical significance of the tests were assessed for each population across all loci by the Wilcoxon signed-rank test available in Bottleneck [31].

To identify the population genetic structure of *R. tanezumi*, two Bayesian model-based cluster analyses were contrasted: Structure [32] and Geneland [33]. Structure 2.3.4 [32] was first used to infer the number of clusters (*Κ*) in the dataset without prior information of the sampling locations. A model in which allele frequencies correlated within populations was assumed (λ was set at 1, the default value). The software was run with the option of admixture, allowing for some mixed ancestry within individuals, and α was allowed to vary. We used 20 independent runs for each value of *Κ* (*K* = 1 to 10), with a burn-in period of 50,000 iterations and 250,000 replications. The method of Evanno et al. [34] was used to determine the most likely number of clusters. This approach uses an ad hoc quantity, Δ*K*, based on the second-order rate of change of the likelihood function between successive values of *K*. The results of 20 replicate runs for each value of *K* were combined using the Greedy algorithm of Clumpp 1.1.1 [35], and summary outputs for each value of *K* were displayed graphically using Distruct v1.1 [36]. Differentiation among clusters was estimated with Wright’s *F*-statistics [37] computed in Fstat according to Weir and Cockerham [38]. Pairwise *F*_*ST*_ for statistical significance was also tested in Fstat to evaluate genetic differentiation between populations. The number of putative migrants (*N*_*m*_) per generation between populations was estimated from pairwise *F*_*ST*_, *N*_*m*_ ≈ (1-*F*_*ST*_)/4*F*_*ST*_ [39]. Critical significance levels for multiple testing were corrected using Bonferroni correction [40].

The second method relied on the spatial cluster model implemented in the Geneland package [33, 41]. Following the user’s manual recommendations, the Markov chain Monte Carlo (MCMC) repetitions were set to 100,000, the thinning was set to 100 and the burn-in period was set to 100 (we eliminated the first 100 iterations when the curve was not constant); the number of groups (K) to be tested was set from 1 ± 7. Each individual was assigned to one of K populations (1 ≤ K ≤ 7) based on its multilocus genotypes and spatial coordinates. To confirm that the run was long enough, we obtained 10 different runs and compared the parameter estimates (K, individual population membership, maps). The best result was chosen based on the highest average posterior density.

The Neighbour-Joining (NJ) tree was computed with Powermarker 3.25 [42], based on microsatellite Nei’s genetic distance [43]. The robustness of population trees was evaluated by bootstrapping over loci. One thousand bootstrapped trees were summarized to obtain a consensus tree, using the Consens module in the Phylip package [44], followed by editing with Treeview [45].

### Mitochondrial DNA analysis

In total 502 individuals were used to explore sequence polymorphism in the mitochondrial COI gene and D-loop region. The mitochondrial COI gene was used not only in population genetics analysis bust also as DNA barcoding at first in specimens identification. DNA samples were used as templates to amplify a 702-bp fragment of COI and a 546-bp fragment of D-loop according to Robins et al. [12]. PCR products were sequenced directly in two directions with an ABI 3100 automatic sequencer (Perkin-Elmer, Waltham, Massachusetts) using the ABi PRISM BigDye Terminator Cycle Sequencing Ready Reaction Kit with AmpliTaq DNA polymerase (Applied Biosystems, Foster City, California).

Sequences were aligned using Muscle [46] within MEGA 6.0 [47]. Basic sequence statistics, including the number of segregating sites (*S*), the number of haplotypes per sample (*H*_*p*_), haplotype diversity (*H*_*p*_*D*), the average number of nucleotide differences (*K*) and nucleotide diversity (*π*) were computed with DnaSP v5 [48]. Neutrality tests used the statistics from the Tajima test, Fu and Li test and Fu test in DnaSP v5.

The phylogenetic relationships of mitochondrial sequences and haplotypes were performed using Bayesian inference (BI), maximum likelihood (ML) and median-joining network (MJ) methods as implemented in Mrbayes 3.2.5 [49], Raxmlgui 1.5 [50], and Network 4.6.1.3 [51], respectively. For the mitochondrial COI sequence, all reported sequences of *R. tanezumi* in Genbank [52–55] (Additional file 2: Table S2) were downloaded and added to the dataset for analysis. COI sequences of *Rattus rattus* (KY754543, JQ668025), *R. pyctoris*(JF499340), *R. argentiventer*(HM217484), *R. exulans* (KC617851), *R*. *andamanensis* (KY605368), *R. norvegicus* (FQ211257, FQ228470), *R. nitidus* (KR996517, JQ918374) and *Mus musculus* (AB444046, KC617843) were added into dataset, and *Cricetulus migratorius* (KU182948, KU182947, KJ466858, JX962138, JX962139) was set as outgroup in the construction of ML and BI trees.

## Results

### Microsatellite analysis

#### Genetic diversity

A total of 502 individual rats collected in this study from 18 populations were successfully genotyped at all 9 microsatellite loci. All loci were polymorphic, showing the number of alleles (*N*_*a*_) as 21 (Rt1), 26 (Rt2), 27 (Rt3), 33 (Rt4), 16 (Rt5), 19 (Rt6), 26 (Rt7), 30 (Rt8) and 24 (Rt9) (Additional file 3: Table S3). The average number of alleles per locus ranged from 9.837 (Rt1) to 14.682 (Rt8). The minimum mean number of alleles of all loci was recorded for the NYC population from Tibet (6.56) and the maximum in the RUL population from Yunnan (14.67). For allele richness, the maximum *R*_*s*_ in RUL (12.353) was almost twice the minimum *R*_*s*_ in the LHS population from Tibet (6.14). The observed heterozygosity (*H*_*o*_) for each locus ranged from 0.28 (Rt4) to 1.00 (Rt9); the minimum *H*_*o*_ was in LHS (0.58) and the maximum *H*_*o*_ in CHQ (0.82) (Table 2). The expected heterozygosity (*H*_*e*_) for each locus ranged from 0.37 (Rt1) to 0.94 (Rt4); the minimum *H*_*e*_ was in LHS (0.67) and the maximum *H*_*e*_ in RUL (0.91) (Table 2). The same situation also occurred in *N*_*e*_ and *I*: the minimum value was found in LHS of Tibet and the maximum value was in RUL of Yunnan. Private alleles were mainly detected in the LIH, QNJ, BAS and RUL populations and were not found in SJZ, XIS, SHH, CHQ, SIC, NYC, LHS and XIY populations. Student’s *t*-Test showed that the mean values of *H*_*e*_ and *R*_*s*_ for Yunnan’s and other region’s populations had statistically significant differences (*H*_*e*_: *P =* 0.022; *R*_*s*_: *P =* 0.005). In general, populations in Yunnan showed a higher genetic diversity than other regions, while the genetic variability of populations in the Tibetan areas and SJZ was low.

**Table 2:** Genetic variation in 18 populations averaged over 9 microsatellite loci: number of alleles per population (*N*_*a*_), effective allele (*N*_*e*_), Shannon’s information index (*I*), allele richness (*R*_*s*_), observed heterozygosity (H_*o*_), expected heterozygosity (*H*_e_), inbreeding coefficient (*F*_*IS*_), mean values for the M-ratio test (M) and *p*-values for the genetic bottleneck detection using the Wilcoxon signed-rank test under Infinite Allele (IAM), Step-Wise Mutation (SMM) and Two-Phase Mutation (TPM) models

	LIH	RUL	BAS	XGL	QNJ	LOY	LEZ	WZS	QUZ	SHH	CHQ	XIS	CHS	SJZ	XIY	SIC	NYC	LHS
*N*_*a*_	14.11	14.67	12.44	10.00	12.67	12.44	11.56	11.33	9.78	10.56	12.00	10.00	10.00	8.00	9.33	8.67	6.56	7.00
*Ne*	8.39	9.53	7.69	6.66	7.86	7.92	7.37	6.76	6.13	5.96	7.28	6.19	5.35	4.69	6.22	5.34	3.96	3.37
*I*	2.33	2.43	2.23	2.05	2.23	2.25	2.16	2.06	1.94	1.97	2.13	1.96	1.89	1.70	1.94	1.81	1.52	1.42
*R*_*s*_	11.68	12.35	10.66	10.00	10.64	10.98	10.74	9.37	8.73	8.611	9.76	8.502	9.202	7.715	9.33	8.06	6.21	6.14
*H*_*e*_	0.89	0.91	0.88	0.87	0.88	0.89	0.88	0.85	0.83	0.824	0.86	0.831	0.819	0.782	0.84	0.80	0.74	0.67
*H*_*o*_	0.72	0.64	0.61	0.69	0.63	0.73	0.78	0.75	0.81	0.769	0.82	0.815	0.725	0.716	0.76	0.72	0.64	0.58
*F*_*IS*_	**0.20**	**0.29**	**0.31**	**0.20**	**0.28**	**0.18**	**0.11**	**0.12**	0.02	0.067	0.05	0.019	**0.115**	0.084	0.09	**0.10**	**0.15**	**0.13**
*M*	**0.38**	**0.33**	**0.32**	**0.34**	**0.37**	**0.40**	**0.35**	**0.37**	**0.44**	**0.44**	**0.42**	**0.41**	**0.38**	**0.38**	**0.39**	**0.41**	**0.38**	**0.36**
*IAM*	**0.001**	**0.001**	**0.001**	**0.003**	**0.001**	**0.001**	**0.005**	**0.001**	**0.001**	**0.001**	**0.001**	**0.002**	0.082	0.082	**0.010**	**0.019**	**0.002**	0.285
*SMM*	0.918	0.248	0.752	0.545	0.674	0.715	0.787	0.918	0.787	0.986	0.82	0.545	0.999	0.981	0.85	0.545	0.82	0.99
*TPM*	0.285	**0.005**	0.064	0.125	0.082	**0.014**	0.326	0.18	**0.024**	0.064	0.125	0.064	0.715	0.248	0.326	0.285	0.285	0.898

Bold characters denote a significant heterozygote deficiency (*p* < 0.05) after correction for multiple testing by the sequential Bonferroni procedure

Some specimens failed to amplify at one locus while succeeded at the remaining loci, suggesting the presence of null alleles. Null alleles were present in only a few populations for three loci, there were one individual at locus Rt6, two individuals at locus Rt5 and four individuals at locus Rt7. To determine whether the null alleles impacted the population genetic analyses, we performed these analyses both before and after the dataset were adjusted for estimated null allele frequencies. The effect of this treatment was minimal and did not significantly change the degree or statistical significance of the estimated parameters.

#### Hardy-Weinberg equilibrium and linkage disequilibrium

The Hardy-Weinberg exact tests were performed at nine loci. No locus was in HWE for all of samples assayed. However, at the population level, 28 out of 162 (17.28%) comparisons did not conform to Hardy-Weinberg expectations after sequential Bonferroni correction, and the deviations were associated with a positive inbreeding coefficient (*F*_*IS*_), reflecting heterozygosity deficits. The populations in Yunnan (Southwest China) had the highest number of loci in departure from HWE (21 out of 28), LOY sample had 3 loci in departure from HWE, WZS sample had 2 loci in departure from HWE, SHH and LEZ populations had 1 locus in departure from HWE respectively (Table 3).

**Table 3:** Probability of assignment of individuals to each population cluster with Bayesian analysis in Structure

Populations	Probability of assignment of individuals to each cluster			Number of loci in departure from HWE
	I	II	III	
LIH	0.9622	0.0288	0.0090	3
RUL	0.9490	0.0290	0.0220	6
BAS	0.9178	0.0542	0.0280	5
XGL	0.8650	0.1130	0.0220	2
QNJ	0.8715	0.0875	0.0410	5
LOY	0.4462	0.5232	0.0307	3
LEZ	0.3416	0.6053	0.0531	1
WZS	0.1645	0.7822	0.0533	2
QUZ	0.0765	0.8724	0.0512	0
SHH	0.0310	0.9418	0.0272	1
CHQ	0.0340	0.8588	0.1072	0
XIS	0.0150	0.8873	0.0977	0
CHS	0.0380	0.8936	0.0685	0
SJZ	0.0278	0.9202	0.0520	0
XIY	0.0522	0.9096	0.0382	0
SIC	0.0282	0.7016	0.2703	0
NYC	0.0130	0.0578	0.9292	0
LHS	0.0090	0.0208	0.9702	0

Fisher’s exact tests were conducted for linkage disequilibrium within all populations. Only 4 pairs out of 648 comparisons (0.62%) were at linkage disequilibrium after sequential Bonferroni correction, and all three such pairs were found in SHH and LOY populations (Rt1/Rt3, Rt8/Rt9, Rt1/Rt8). No pair of loci appeared in LD in more than one population, suggesting genetic independence between loci.

#### Demography

Tests for genetic bottlenecks yielded mixed results (Table 2). The *M* ratio was low and indicated historical bottleneck in all populations. Significant deviations from mutation-drift equilibrium conditions under the IAM model were found in all populations except the SJZ, CHS, and LHS populations. In contrast, the test for each population gave non-significant results under the SMM model. According to the TPM model, the RUL population showed heterozygote excess in both percentages of stepwise mutations, and the results for the LOY and QUZ populations suggested a heterozygosity excess only in 80% of stepwise mutations, which indicated that bottlenecks would have occurred recently under pressure of rodent control in these populations.

#### Genetic differentiation and gene flow

**Fig. 2:**
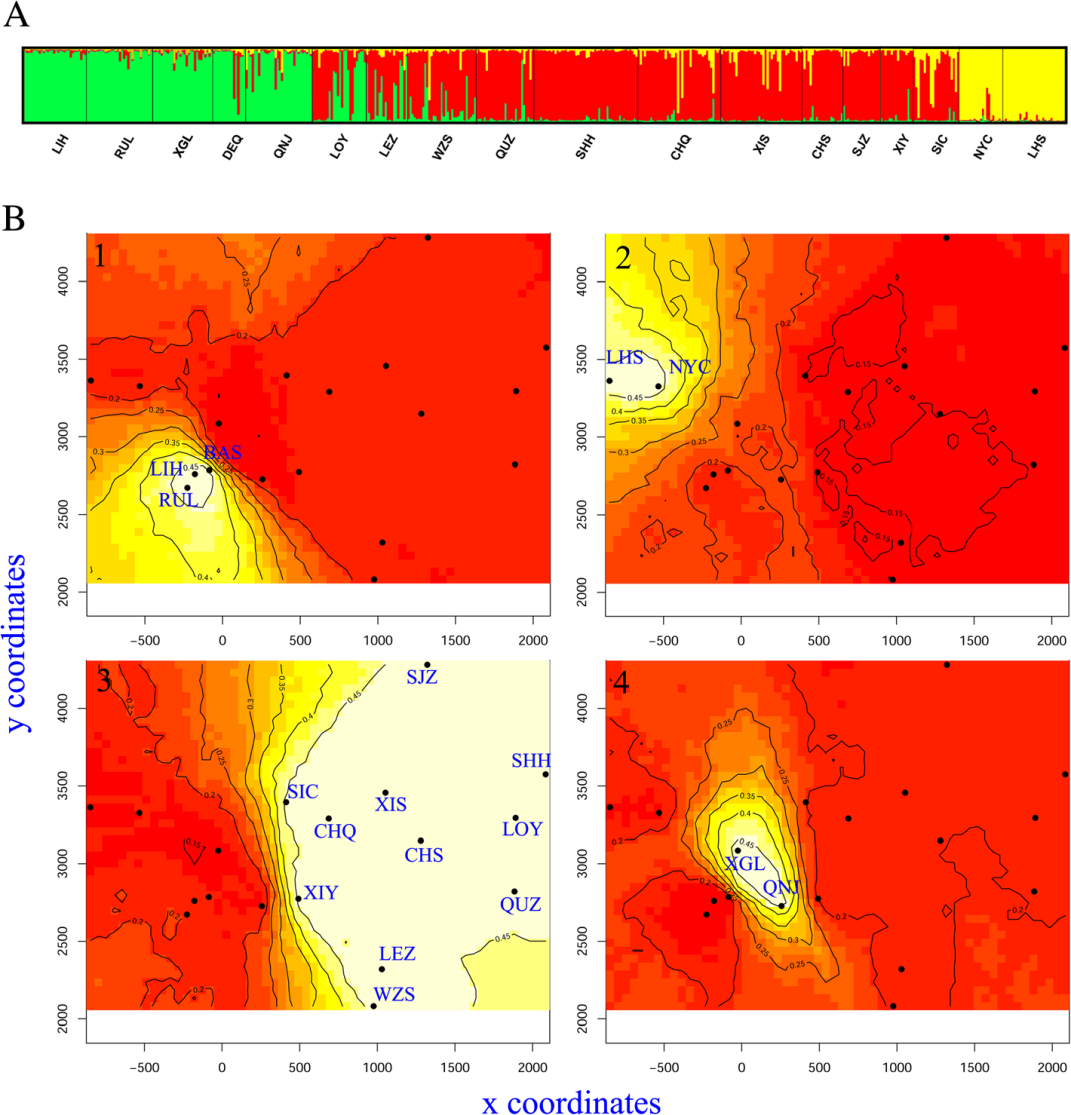
*Rattus tanezumi* population structure by nine microsatellite loci in 18 populations. (**a**) Bayesian assignments using STRUCTURE 2.3.4 (K = 3, with the ΔK likelihood [34]). Individuals are represented by thin vertical bars coloured according to inferred group membership, with red representing cluster I, yellow areas presenting cluster II and green for cluster III. (**b**) Maps of the posterior probabilitis to belong to genetic groups inferred in Geneland. Color gradient represents high (white) to low (red) posterior probabilities

The significant deviations from HWE with heterozygote deficiency and the presence of linkage disequilibrium suggest the presence of population subdivision within the samples (the Wahlund effect). Bayesian analysis divided all individuals into three clusters (posterior probability of Bayesian clustering Ln (D) likelihood score optical for *K* = 3). Most individuals of LIH, XGL, QNJ, BAS and RUL populations belonged to Cluster I; almost all individuals of WZS, QUZ, SHH, CHQ, XIS, CHS, SJZ, XIY, and SIC populations were of Cluster II; and individuals of NYC and LHS were members of Cluster III. While populations of LOY, LEZ were composed with individuals of clusters I and II (Table 3 and Fig. 2a), which indicated the coexistence of two gene pools in these two populations.

Moderate levels of genetic differentiation were found between populations. The overall *F*_*ST*_ across all loci was 0.064 and was highly significant (*P* < 0.001). We then tested the genetic heterogeneity among populations within and between the clusters. We chose populations in which more than 70% of individuals were assigned to cluster I or II for analysis. Cluster I included 5 populations (LIH, XGL, QNJ, BAS and RUL), while cluster II included 9 populations (QUZ, SHH, CHQ, XIS, CHS, SJZ, and XIY). Within each cluster, pairwise *F*_*ST*_ was moderate: 0.020 (CHQ-CHS) to 0.077 SHH-SIC) in cluster I and 0.022 (LIH-RUL) to 0.048 (LIH-XGL) in cluster III. Among the clusters, a higher level of differentiation was demonstrated, with *F*_*ST*_ from 0.051 (QUZ-QNJ) to 0.168 (LIH-LHS). *N*_*m*_ estimates among populations were higher (2.980 to 12.005) within clusters and lower (1.24 to 4.49) among clusters (Additional file 4: Table S4).

Analysis using Geneland yielded a modal number of populations between 2 and 7, with a higher proportion of K = 4 (posterior probability = 0.52). The run with the highest average posterior density was selected (− 14,771.26). Cluster 1 included three populations from Southwest of Yunnan (RUL, BAS and LIH), and cluster 4 included two populations from North of Yunnan (XGL and QNL). Cluster 2 was constituted by populations from Tibet (LHS and NYC). And cluster 3 was composed of other populations from south, east and middle of China (Fig. 2b).

Tests of isolation by distance were performed for each population cluster and for all the populations together. For all populations, weak correlations were detected between genetic and geographic distances (*R*^2^ = 0.111, *P* = 0.025), while for cluster I and II, the correlations were stronger (*R*^*2*^ *=* 0.527, *P <* 0.01). The correlations for cluster III were not significant (*R*^*2*^ = 0.209, *P* = 0.17). These results suggest that geographic distance weakly contributes to the genetic differentiation in *R. tanezumi* populations.

The unrooted NJ dendrogram based on Nei’s distance depicted the structure of population differentiation (Fig. 1b). It demonstrated that the populations of Yunnan clustered first and then showed a close relationship with populations from coastal areas (LOY of Zhejiang, LEZ of Guangdong, WZS of Hainan, and QUZ of Fujian). At the other end of the tree were populations from Central China and Tibet (LHS and NYC).

### DNA sequencing analysis

#### MtDNA polymorphism and divergence

Nucleotide sequences of mtDNA COI (702 bp) and D-loop (546 bp) were obtained for 502 *R. tanezumi* specimens from 18 populations. Overall, the polymorphism in the COI region was moderate, with 37 segregating sites defining 38 haplotypes. The nucleotide diversity indices of the XGL population from Yunnan were the highest (*HpD* = 0.842, *K* = 3.458, *π* = 0.00493), while the lowest indices occurred in the WZS population from Hainan (*HpD* = 0.061, *K* = 0.061, *π* = 0.00009). The SHH and LHS populations from Shanghai and Tibet also showed lower genetic diversity. The most frequent haplotype, H1 (48.1%), was detected in all geographic populations except LIH and RUL. For the D-loop region, the polymorphism was much higher than in COI. There were 60 segregating sites defining 70 haplotypes. The highest nucleotide diversity indices were similarly among the QNJ population in Yunnan (*HpD* = 0.905, *K* = 11.315, *π* = 0.02076), while the lowest indices occurred in the XIS population from Hubei (*HpD* = 0.148, *K* = 0.151, *π* = 0.00028) (Table 4). Populations in Tibet (NYC and LHS) clearly manifested low genetic polymorphism compared with other regions. In common with the COI results, the most frequent D-loop region haplotype, H1 (44.5%), was detected in all geographic populations but was absent from LIH and RUL, as well as BAS. Tajima’s D, and Fu & Li’s statistics were negative for both genes (*D* = − 1.724, D* = − 1.722 and F* = − 2.088 for COI; *D* = − 0.288, D* = − 0.334 and F* = − 0.376 for D-loop) but not statistically significant, while Fu’s Fs statistic was significantly negative (*Fs* = − 26.381, *P* < 0.001 for COI; *Fs* = − 22.993, *P* < 0.001 for D-loop).

**Table 4:** Summary statistics for COI and D-loop polymorphisms in all populations

Populations	mtDNA	N	*S*	*Hp*	*HpD*	*K*	*π*
LIH	COI	29	6	6	0.719	1.867	0.00266
	D-loop	30	18	8	0.805	5.421	0.00995
RUL	COI	32	14	11	0.792	2.454	0.00350
	D-loop	31	36	10	0.763	10.340	0.01894
BAS	COI	30	8	6	0.680	1.561	0.00222
	D-loop	29	27	11	0.884	6.202	0.01138
XGL	COI	16	10	6	0.842	3.458	0.00493
	D-loop	16	35	8	0.875	11.725	0.02147
QNJ	COI	32	8	6	0.800	2.952	0.00420
	D-loop	32	32	10	0.905	11.315	0.02076
LOY	COI	25	10	7	0.707	2.240	0.00319
	D-loop	26	25	5	0.600	6.689	0.01227
LEZ	COI	20	7	3	0.484	1.474	0.00210
	D-loop	20	19	3	0.595	4.005	0.00735
WZS	COI	33	1	2	0.061	0.061	0.00009
	D-loop	33	3	4	0.563	0.727	0.00133
QUZ	COI	28	5	5	0.434	0.762	0.00011
	D-loop	28	12	4	0.585	1.304	0.00239
SHH	COI	50	4	3	0.153	0.310	0.00044
	D-loop	50	10	4	0.424	0.762	0.00139
CHQ	COI	40	2	2	0.296	0.592	0.00084
	D-loop	40	14	8	0.669	3.212	0.00603
XIS	COI	39	2	3	0.452	0.467	0.00067
	D-loop	39	2	3	0.148	0.151	0.00028
CHS	COI	18	1	2	0.366	0.366	0.00052
	D-loop	20	2	3	0.616	0.711	0.00130
SJZ	COI	18	1	2	0.523	0.523	0.00074
	D-loop	18	5	5	0.680	1.379	0.00253
XIY	COI	16	2	3	0.575	1.025	0.00146
	D-loop	16	17	5	0.792	6.050	0.01110
SIC	COI	22	4	4	0.567	0.961	0.00137
	D-loop	22	11	4	0.455	3.043	0.00557
NYC	COI	21	1	2	0.381	0.381	0.00054
	D-loop	21	1	2	0.381	0.381	0.00070
LHS	COI	30	1	2	0.331	0.331	0.00047
	D-loop	30	1	2	0.331	0.331	0.00061
Overall	COI	499	37	38	0.747	2.016	0.00287
	D-loop	501	60	70	0.794	7.928	0.01455

#### Phylogenetic analysis

**Fig. 3:**
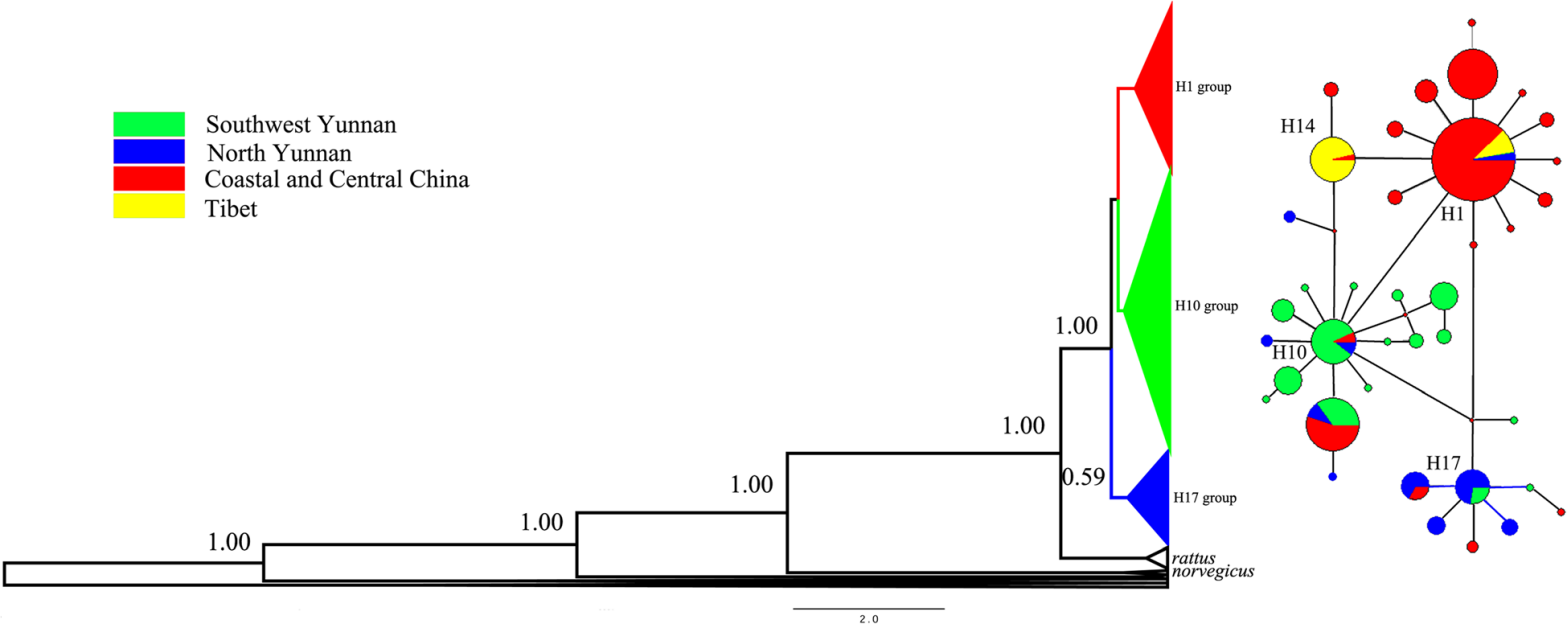
Haplotype BI phylogram (**a**) and networks (**b**) based on COI sequences. The size of the pie charts represents the number of individuals who possess the haplotype, and the colours represent the regions where those individuals were collected. Each connecting line represents one mutational step between the different haplotypes

The Median-Joining Networks show a significant geographical component to variation. For COI, the network shows three distinct haplogroups. Haplotypes from non-Yunnan regions form a star-like pattern centred at H1, and haplotypes from southwest and northeast regions of Yunnan form two star-like patterns centred around H10 and H17, respectively. Furthermore, H4 (only found in LEZ) and H24 (found in both QNJ and LOY) are most closely related to centre H17 (Fig. 3b, Additional file 5: Table S5 for details). The structure of the network for D-loop (Additional file 5: Table S5) is approximately equivalent to that for COI, but the network is more complicated.

For the mitochondrial COI data, the BI analysis indicated that the whole haplotypes, including haplotypes of reported COI sequences, were separated into three middle supported clades (Fig. 3a). These clades were named after the three centre haplotypes of the networks (Fig. 3b). The H1 and H10 clades were more closely related than to the h17 clade (Fig. 3a). These reported sequences from Southeast Asia and Africa did not share haplotypes with sequences of present research.

## Discussion

### Population genetics of *R. tanezumi*

In Yunnan *R. tanezumi* is controlled almost every year in either urban or rural areas because it is host of *Yersinia pestis*, the pathogen of bubonic plague. As a result, the high departure from HWE were observed in all populations from Yunnan, which indicated a population subdivision (Table 3). The results of genetic bottlenecks test (M ratio test and IAM modle) also suggested that bottleneck occurred in most of populations after control with high pressure.

*Rattus tanezumi* population was first found at Shijiazhuang of Hebei at 2004 [9]. The genetic diversity of the population in present research showed that SJZ population and Tibet populations had lower genetic variability (*Rs* and *He* in Table 2). Because of the short colonization history of SJZ population, the low genetic diversity could be attributed to the founder effect. The low genetic diversity of populations from Tibet (LHS and NYC) could only be explained as a result of the founder effect as there were no rodent control measures in Tibet. The recent demographic expansion of these populations was also supported with the results of Tajima’s D, and Fu & Li’s statistics.

### The origin area of *R. tanezumi*

Two different analysis methods based on microsatellites data showed different results. According to the result with Structure, all samples of *R. tanezumi* could be divided into three clusters, and populations collected from Yunnan belonged to cluster III. While the results of Geneland divided populations of Yunnan into two groups, the first including three populations from Southwest Yunnan and the second including two populations from North Yunnan, which was concurrent with the results of haplotype network based on mitochondrial COI sequence. The haplotype network of COI shows two haplotype groups from Yunnan, one from Southwest and another from North Yunnan (Fig. 3b, Additional file 5: Table S5 for detail). The haplotype group from North Yunnan were scarce in other populations.

Populations of *R. tanezumi* from Yunnan showed distinctive characteristics in this study: first, they had many alleles in microsatellite analysis (average *N*_*a*_ for five populations was 12.78, Table 2); second, they had a high number of haplotypes in mtDNA analysis (Table 4). These characters mean that the populations of *R. tanezumi* from Yunnan have high genetic diversity compared with populations from other areas of China.

Yunnan is located at the border area of Southwest China and Southeast Asia. These areas are special regions with various habitats both for aquatic and terrestrial organisms [56–59] because of the intricate geological and climatic history and were therefore regarded as two hotspots of high biodiversity and endemism [60]. In these areas, the young uplifting plateau (Tibetan Plateau), the old eastern escarpment and the southeast slope (Hengduan Mountains) yield highly heterogeneous habitats not only to ‘living fossil’ species, such as the Laotian rock rat *Laonastes aenigmamus* [61] but also to some new speciated species, for example *Apodemus ilex* [62]. Besides the species level diversity, high intraspecific genetic diversity of some species in these areas was also frequently reported [63, 64] because these areas were refuges for different species during glacial periods. According to Musser and Carleton [65], *R. tanezumi* is indigenous to the north of the India subcontinent, South and Central China, and mainland Indochina in which Yunnan is the central area. Therefore, according to the genetic diversity of Yunnan populations of *R. tanezumi*, the location and the biodiversity background of Yunnan, it could be assumed that Yunnan and the adjacent area of Southeast Asia could be regarded as the original centre of *R. tanezumi*.

### The expansion route of *R. tanezumi* into mainland China

Although Yunnan is adjacent to Guizhou, Sichuan and Tibet, the rat populations from these provincial areas do not show a close relationship (Additional file 4: Table S4). The microsatellite analysis shows that there is no close relationship between populations of Yunnan, and those from Guizhou, Sichuan and Tibet (Fig. 1b and Fig. 2). The haplotype analysis of mtDNA sequences shows that the populations of Yunnan have a high diversity of haplotypes (H10 and H17 groups in Fig. 3), while populations from these three provincial areas share only a few haplotypes (H1 group). Populations from Tibet show only two haplotypes of COI and lack any particular commonality with Yunnan haplotypes (Fig. 3b), although the geographic distance between Tibet and Yunnan is very short (Fig. 1a). This result suggests that *R. tanezumi* in Yunnan did not spread to Tibet naturally or with the help of caravan transportation.

The unrooted NJ dendrogram based on genetic distance demonstrated a visualized relationship of populations from different areas of China (Fig. 1b). Based on the relationship, the early expansion of *R. tanezumi* in China could be inferred as having two steps: the first step as spreading through coastal areas via ocean-based shipping transportation, and the second as expanding from the coastal areas into Central China via shipping along the Yangzi River. Aplin *et a.l* reported that *R. tanezumi* expanded in West Pacific with the diaspora of Austronesian-speakers about 4000 years ago [66], which supported the possibility of expanding with ocean shipping transportation in that period. Because the populations of Tibet are closely related to the populations of Sichuan, the sources of the Tibetan populations are presumed to be Sichuan instead of Yunnan, and the route of expansion was likely the modern Chuan-Zang Highway instead of the Tea Horse Ancient Road.

Data of allelic patterns across populations showed in detail that three populations (SJZ, LHS and NYC) have a low number of alleles and effective alleles (*N*_*a*_ and *N*_*e*_) compared with other populations, with the two populations from Tibet (NYC and LHS) having the lowest values (Table 2). These data supported that the populations from Tibet are newly imported and, as a result, suffer from the founder effect and lose genetic diversity during importation and colonization. The population from Hebei (SJZ) also has few alleles and effective alleles, and was only higher than those of populations from Tibet (Table 2). These data reveal that the SJZ is also a newly colonized population with low genetic diversity. As *R. tanezumi* was found in Tibet before 1995 [8], and this species was not reported in Hebei until the 2000s, SJZ population could be considered a young population compared with the population from Tibet. The relative high genetic diversity of SJZ compared with that of NYC and LHS suggests that there were more individuals imported to Hebei from South and Central China in a short time period through the complex modern transportation system. The convenient transportation systems between North and Central China give more chances for *R. tanezumi* to expand to North China [67, 68] than to West China.

All reported COI sequences in Genbank were added in the phylogeny analysis. Most of these sequences came from samples of Southeast Asia, such as Thailand and Vietnam [52, 54, 55]. The result showed that the haplotypes of these sequences belonged to the three haplotype groups (Additional file 2: Table S2). And these sequences did not share haplotype with sequences from China. This result suggested that there was no genetic exchange between populations from sampling areas of China and Southeast Asia, although there were imported individuals intercepted from international cargo ships from different ports of China [69].

### The dispersal ability of *R. tanezumi* and other *Rattus* species

The Mantel test for IBD presented interesting results. The dispersal pattern of *R. tanezumi* in Yunnan does not fit IBD, while populations in other areas of China show an obvious IBD pattern. Because of the complex landscape, the dispersal of *R. tanezumi* in Yunnan should be restricted by geographic barriers, such as valleys and mountains, and thus could not fit IBD. The dispersal of *R. tanezumi* in other areas was also expected to lack IBD because *R. tanezumi*, as a typical commensal rat, can use human transportation to achieve long-distance dispersal. However, the Mantel test shows a strong pattern of IBD for these populations. This result suggests that long-distance dispersal events were rare in the historical expansion of *R. tanezumi* in China, even though this species is widely distributed in China. However, in recent decades, the northward expansion of *R. tanezumi* has become obvious in North China, such as in Shanxi and Qinghai [11, 68], which suggests that modern, quick transportation has begun to give more opportunities to *R. tanezumi* in long-distance dispersal.

Some species of *Rattus* are regarded as commensal species, including *R. rattus*, *R. norvegicus*, and *R. tanezumi*. These species are all able to utilize human-made environments and transportation tools for survival and expansion. However, when two *Rattus* species are analyzed together for their commensal ability, there are differences between the different species. *R. rattus* of the Western Ghats of India shows a stronger dispersal ability compared with *R. satarae*, a native species of the southwest Indian Peninsula [70]. While *R. rattus* in the United States shows low long-distance dispersal ability when it competes with *R. norvegicus* [71]. In China *R. norvegicus* and *R. tanezumi* are two important commensal rodents. The former occurs mainly in southern China, and the latter is recorded in all provincial areas except Tibet [72]. In the last half-century, *R. norvegicus* invaded Xinjiang along the railway and expanded through rural areas of Xinjiang with the help of modern transportation [19, 73]. *R. tanezumi* has been reported in some provinces in North and Northwest China in recent decades [11, 68]. Compared to the northwestward expansion of *R. norvegicus* in China, *R. tanezumi* shows a low speed in spreading northward in recent years. This phenomenon suggests that *R. tanezumi* is not as fit as *R. norvegicus* for long-distance dispersal using modern transportation systems.

## Conclusion

*R. tanezumi* populations in mainland China originated from Yunan and adjacent Southeast Asia areas. The ancient trade in Southwest China based on caravans and the Tea Horse Ancient Road did not affect the range expansion of *R. tanezumi* in this area. The early dispersal of this species depended on shipping transportation over the sea and then expanded from coastal areas into Central China along the Yangzi River. The populations of Tibet were imported from Sichuan in the modern era via the Chuan-Zang highway rather than along the Tea Horse Ancient Road.

## Additional files

Additional files 1: **Table S1**. Microsatellite loci of *R. tanezumi* and primers used in this research. (XLSX 10 kb)

Additional files 2: **Table S2**. List of haplotypes used in the phylogram of Fig. 3, haplotype 39–129 were found in downloaded sequences from Genbank. (XLSX 14 kb)

Additional files 3: **Table S3**. Genetic variability at nine microsatellite loci in *R. tanezumi* populations from China. (XLSX 17 kb)

Additional files 4: **Table S4**. Pairwise genetic distance (FST) and gene flow (Nm) for 15 populations based on microsatellite data. (XLSX 11 kb)

Additional files 5: **Table S5.** The haplotype networks of COI and Dloop sequences. (XLSX 64 kb)

Additional files 6: **Figure S1.** STR data. (TIF 6056 kb)

## Abbreviations

HWE: Hardy-Weinberg equilibrium
IAM: Infinite Allele Model
IBD: Isolation by distance
LD: Linkage disequilibrium
MCMC: Markov chain Monte Carlo
NJ: Neighbour-Joining
SMM: Stepwise Mutation Model
TPM: Two-Phase Model
