# Supplementary figures and images for: Dispersal route of the Asian house rat (Rattus tanezumi) on mainland China: insights from microsatellite and mitochondrial DNA
Source: BMC Genet. 2019 Jan 22;20:11. doi: 10.1186/s12863-019-0714-3 (PMC6341715; doi:10.1186/s12863-019-0714-3)

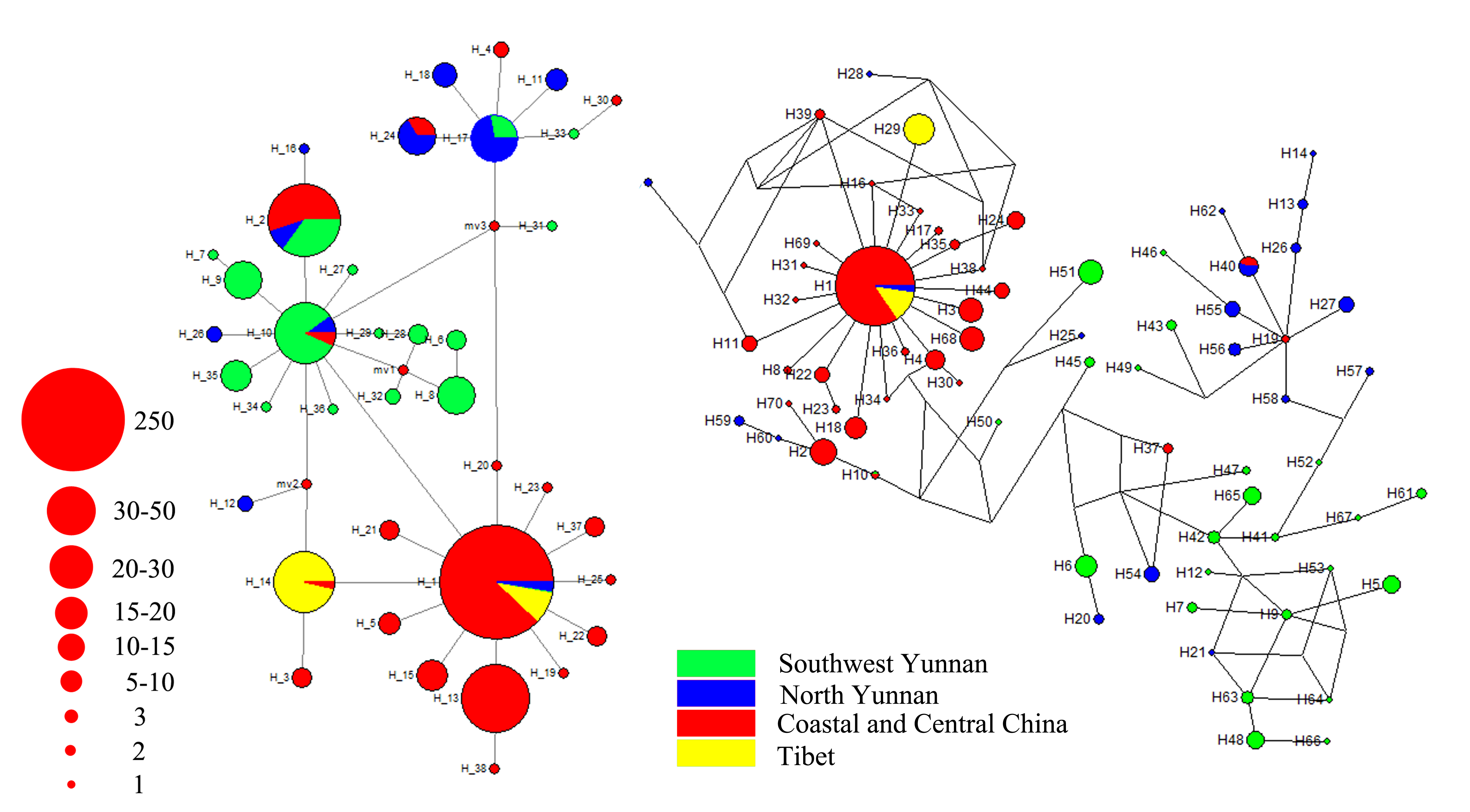

Supplement: Supplementary file 6 — Figure S1. STR data. (TIF 6056 kb) [file 12863_2019_714_MOESM6_ESM.tif]
